# C/MIC > 4: A Potential Instrument to Predict the Efficacy of Meropenem

**DOI:** 10.3390/antibiotics11050670

**Published:** 2022-05-16

**Authors:** Yichang Zhao, Chenlin Xiao, Jingjing Hou, Jiamin Wu, Yiwen Xiao, Bikui Zhang, Indy Sandaradura, Hong Luo, Jinhua Li, Miao Yan

**Affiliations:** 1 Department of Pharmacy, The Second Xiangya Hospital, Central South University, Changsha 410011, China; zhaoyichang@csu.edu.cn (Y.Z.); xiaochenlin@csu.edu.cn (C.X.); houjingjing2020@csu.edu.cn (J.H.); wujiamin8@foxmail.com (J.W.); xiaoyw2005@163.com (Y.X.); bikui_zh@126.com (B.Z.); 2 International Research Center for Precision Medicine, Transformative Technology and Software Services, Changsha 410011, China; 3 School of Medicine, University of New South Wales, Sydney, NSW 2052, Australia; indy.sandaradura@health.nsw.gov.au; 4 Centre for Infectious Diseases and Microbiology, Westmead Hospital, Sydney, NSW 2145, Australia; 5 Department of Respiratory and Intensive Care Unit, The Second Xiangya Hospital, Central South University, Changsha 410011, China; luohong1003@163.com

**Keywords:** meropenem, efficacy, bacterial pneumonia, meropenem concentration, therapeutic drug monitoring

## Abstract

This prospective study aimed to explore the determinants of meropenem trough concentration (C_trough_) in patients with bacterial pneumonia and to investigate the association between its concentration and efficacy. From January 2019 to December 2019, patients with pulmonary infections were prospectively enrolled from the intensive care unit. Factors affecting the meropenem trough concentration were analyzed, and a multiple linear regression model was constructed. Logistic regression analyses were used to investigate the relationship between C_trough_ and clinical efficacy. A total of 64 patients were enrolled, in whom 210 meropenem concentrations were measured. Of the total, 60.9% (39/64) were considered clinically successful after treatment. C_trough_ may increase with increased blood urea nitrogen, albumin, and concomitant antifungal use. By contrast, concentration may decrease with increased endogenous creatinine clearance rate. Six variables, including C_trough_/minimum inhibitory concentration (MIC) > 4, were associated with the efficacy of meropenem. There was an independent correlation between C_trough_/MIC > 4 and efficacy after fully adjusting for confounding factors. Based upon renal function indexes, it is possible to predict changes in meropenem concentration and adjust the dosage precisely and individually. C_trough_/MIC > 4 is a potential instrument to predict successful treatment with meropenem.

## 1. Introduction

Pulmonary infections in critically ill patients are associated with poor outcomes and have attributable mortality of 30–50% [1,2,3,4]. One of the principles of empirical antimicrobial therapy for pneumonia [5] is to cover as many potential pathogens as possible in the early empiric stage of therapy utilizing broad-spectrum antimicrobial agents. In the past few decades, carbapenems have been in widespread clinical use, and are considered as the last line of treatment of gram-negative bacteria infection [6,7,8]. 

Meropenem is a second-generation carbapenem antibiotic agent, which has a broad-spectrum, highly effective activity and complex drug exposure in vivo [9,10]. In terms of clinical applications, meropenem has a good clinical effect on hospital-acquired (nosocomial) severe infections because nosocomial infection is mainly caused by G (−) bacteria. Meropenem remains the preferred choice for definitive treatment of ceftriaxone nonsusceptible Escherichia coli and Klebsiella [11]. Meropenem is characterized by linear pharmacokinetics in vivo, and its elimination half-life in healthy volunteers is about 1 h [12]. Due to the highly abnormal physiology of critically ill patients and the application of various treatment techniques, in vivo exposure of meropenem has been shown to be highly complex. Several population pharmacokinetic studies [13,14] suggest that meropenem shows significant individual variability in concentration level and pharmacokinetic parameters. The susceptibility of pathogenic bacteria (measured by the minimum inhibitory concentration (MIC)) may also be lower in critical illness. Therefore, meropenem exposure may need to be higher in this population to achieve optimal clinical outcomes [15]. It is necessary to optimize the dosing regimen of meropenem with therapeutic drug monitoring (TDM) [16,17,18]. Nevertheless, the factors influencing meropenem concentration in patients with pulmonary infection require further study, and the relationship between the concentration and the efficacy remains incompletely defined. Accordingly, the present study aimed to explore the determinants of meropenem concentration in patients with pulmonary infections and to investigate the association between its concentration and efficacy in a cohort of critically ill patients.

## 2. Results

### 2.1. Patient Characteristics

**Figure 1:**
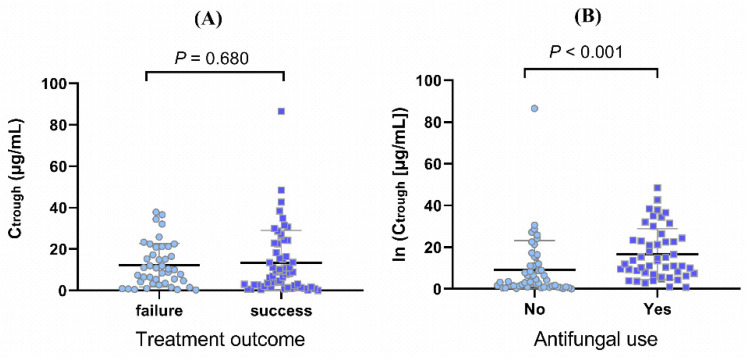
Distinction of meropenem trough concentrations in different groups (*n* = 101); (**A**) treatment outcome and (**B**) concomitant antifungal use.

A total of 64 patients with 210 meropenem concentrations (C) were enrolled in this prospective study. The demographic parameters and primary physiological indicators are listed in Table 1. Briefly, 39/64 concentrations (60.9%) were considered clinically successful meropenem treatments. The concentration of albumin and body temperature of patients were significantly lower in the “clinical success” group. C-reactive protein (CRP), concomitant antibiotic use, concomitant antiviral drug use, and the percentage of lymphocytes showed statistical differences among the two groups. However, there were no statistical differences among groups in terms of meropenem trough concentration (Figure 1A), age, sex, and other factors (listed in Table 1). In the clinical success group, the median age was 65 years and 76.9% were males. The median meropenem concentration was 18.33 μg/mL and the interquartile range was 5.92 μg–33.42 μg/mL.

In addition, 53 (82.81%) patients were given an initial dose of 1000 mg q8h; 10 (15.62%) of them were given an initial dose of 500 mg q8h; and only 1 (1.5%) patient was given an initial dose of 250 mg q8h because of renal insufficiency. All infusion durations were over 1 h. For 76.56% (49/64) of the patients, the infusion duration was 2 h, while for 17.19% (11/64) of them, the infusion duration was 1 h. Only 6.25% of them were treated with infusion times of over 2 h. We collected 139 MIC values on the sampling day. Pathogens detected in the patients comprised Acinetobacter baumanii, Klebsiella pneumoniae, Pseudomonas aeruginosa, Stenotrophomonas maltophilia, Enterobacter cloacae, Alcaligenes spp, Klebsiella oxytoca, Ralstonia pickettii, and Burkholderia cepacia. After treatment, 57.6% (80/139) were of the treatments were considered to be clinically successful, with 86 concentrations (61.9%) reaching target 1 (C/MIC > 3) and 78 concentrations (56.1%) reaching target 2 (C/MIC > 4).

### 2.2. Univariate Analysis of Meropenem trough Concentration

The results of univariate analysis of C_trough_ are displayed in Table 2. Infusion duration, age, concomitant antifungal use (Figure 1B), [Na^+^], albumin, blood urea nitrogen, creatinine, uric acid, procalcitonin, and erythrocyte sedimentation rate showed significant positive linear trends with ln (C_trough_), and the Spearman correlation coefficients (rs) were 0.207 (*p* = 0.038), 0.278 (*p* < 0.001), 0.243 (*p* < 0.001), 0.215 (*p* = 0.002), 0.161 (*p* = 0.019), 0.423 (*p* < 0.001), 0.404 (*p* < 0.001), 0.366 (*p* < 0.001), 0.220 (*p* = 0.001), and 0.218 (*p* = 0.001), respectively. Significant negative correlations were observed between dose (rs = −0.289, *p* = 0.003), hemoglobin (rs = −0.269, *p* < 0.001), red blood cells (RBC, rs = −0.251, *p* < 0.001), alanine aminotransferase (ALT, rs = −0.145, *p* < 0.001), and endogenous creatinine clearance rate (CG−CLCR, rs = −0.499, *p* < 0.001) and ln (C_trough_).

### 2.3. Renal Function Indexes: Determinants of Meropenem trough Concentration

**Figure 2:**
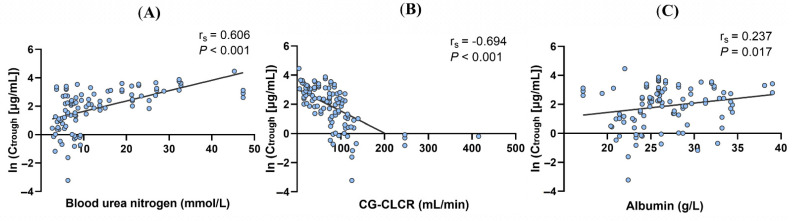
Correlation analysis between ln (C_trough_) and its determinants (**A**) blood urea nitrogen, (**B**) CG-CLCR endogenous creatinine clearance rate and (**C**) albumin.

On the basis of the results of univariate analysis, we constructed a multiple linear regression model using the stepwise method. The final model revealed that infusion duration (coefficient [β] = 0.495; *p* = 0.006), blood urea nitrogen (coefficient [β] = 0.051; *p* < 0.001), CG-CLCR (β = −0.009; *p* < 0.001), and albumin (β = 0.071; *p* = 0.004) were determinants of ln (C_trough_) (Figure 2). Details of the optimal multiple linear regression model are presented in Table 3.

Ln (C_trough_) increased by 0.051 μg/mL and 0.071 μg/mL with a one unit increase in blood urea nitrogen and albumin. The trough concentration (ln) tended to be 0.009 μg/mL lower if CG-CLCR increased by one unit. With a 1 h infusion duration increase, the trough concentration (ln) increased by 0.495 μg/mL. The linear regression equation was as follows:

Ln (C_trough_) = −0.914 + 0.051 × blood urea nitrogen − 0.009 × CG-CLCR + 0.071 × albumin + 0.495 × infusion duration (1)

### 2.4. Diagnosis of the Multiple Linear Model

The fitness coefficient of the final regression equation was R^2^ = 0.531, indicating that these factors explained 53.1% of the variability in the disposition of meropenem. The F-value of final regression was 29.360, with a *p*-value of <0.001, suggesting a linear regression relationship among these factors. In addition, the collinearity of blood urea nitrogen, creatinine clearance, and albumin was diagnosed using the variance inflation factor (VIF), and final factors were not collinear with one another (VIF < 2). Finally, we evaluated the residuals. The residual of the final model established obeyed the normal distribution and conformed to the precondition of the regression equation, suggesting that the final model was stable and reliable.

### 2.5. C_trough_/MIC > 4 Was Associated with Efficacy

Univariate analysis of three groups of meropenem concentration (trough concentration, 2 h concentration, and peak concentration) showed that there were no statistical differences between meropenem concentration in terms of efficacy. Further analysis combined with MIC was performed. Since the trough concentration determination of meropenem monitoring is the most widely used in clinical practice, and the trough concentration accounts for the largest proportion in our samples, the analysis result of trough concentration is mainly shown.

According to the univariate logistic regression analysis of trough concentration group, seven variables, including C_trough_/MIC > 4 (target2), RBC, PCO_2_, and CRP, were associated with the efficacy of meropenem. In contrast, no correlation was observed between C_trough_/MIC > 3 and efficacy in the univariate logistic regression analysis (*p* = 0.051).

**Figure 3:**
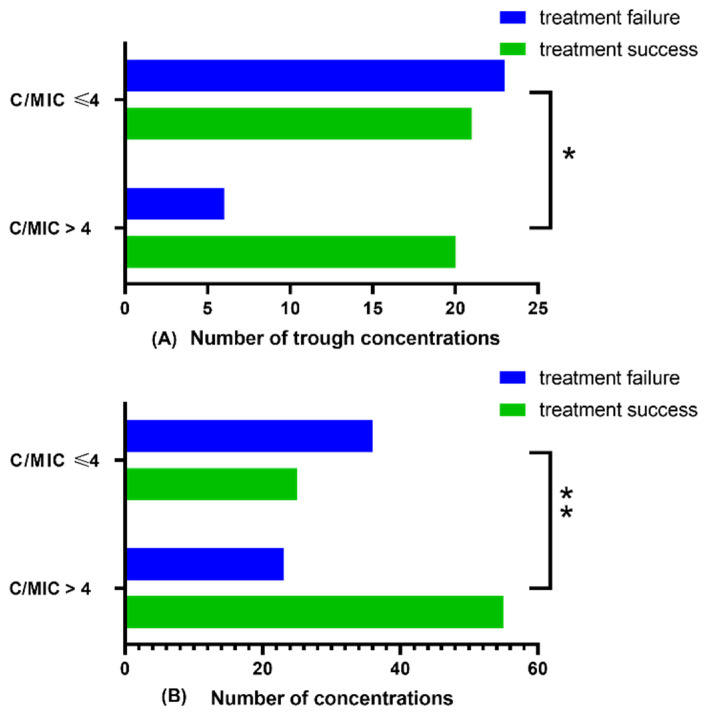
Distinction of treatment outcome in different groups of C_trough_/MIC and C/MIC. (**A**) Number of trough concentrations (*n* = 70), (**B**) Number of concentrations (*n* = 139). C_trough_**,** trough meropenem concentration; C, overall meropenem concentration; MIC, minimum inhibitory concentration; * represents *p* < 0.05, ** represents *p* < 0.001.

**Figure 4:**
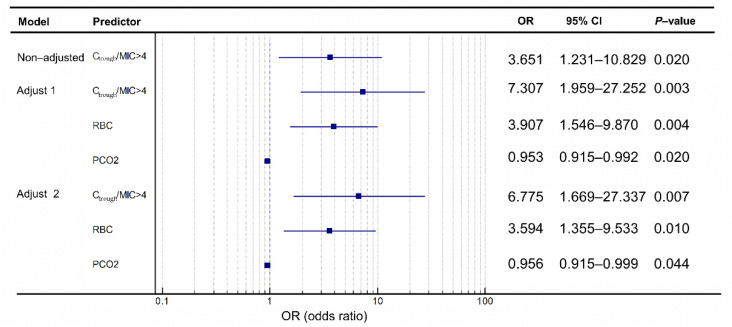
Forest plot of predictors of meropenem treatment success. OR, odds ratio; RBC, red blood cells; PCO_2_, arterial partial carbon dioxide pressure. Model 1: adjusted for RBC and PCO_2_; model 2: adjusted for RBC, PCO_2_, albumin, CRP, and total bile acid.

There was an independent correlation between C/MIC > 4 and efficacy when unadjusted for confounding factors. Correlation was observed between C_trough_/MIC > 4 and efficacy (Figure 3B, *p* < 0.001) and C_trough_/MIC in the chi-square test (Figure 3A, *p* = 0.017). Two more models were constructed after adjusting different confounding factors that simultaneously impact the efficacy, as presented in Figure 4. In the non-adjusted model, the results indicate that the patients whose C_trough_/MIC > 4 were 365.1% more likely to achieve a favorable efficacy outcome than those whose C/MIC ≤ 4. Moreover, the efficacy was significantly different in patients whose C_trough_/MIC > 4 vs. those whose C/MIC ≤ 4 in the three models.

In the fully adjusted model (model 2), a C_trough_/MIC > 4, RBC and PCO_2_ were independent influencing factors for the efficacy (Figure 4). The odds ratio for C_trough_/MIC > 4 was 6.755, implying that for C_trough_/MIC > 4 groups, the probability of a favorable outcome is 6.755 times that of the C_trough_/MIC ≤ 4 group. Therefore, we found that C_trough_/MIC > 4 was a potential predictor of meropenem efficacy, instead of C_trough_/MIC > 3.

### 2.6. Receiver Operating Characteristic Curve Analysis

**Figure 5:**
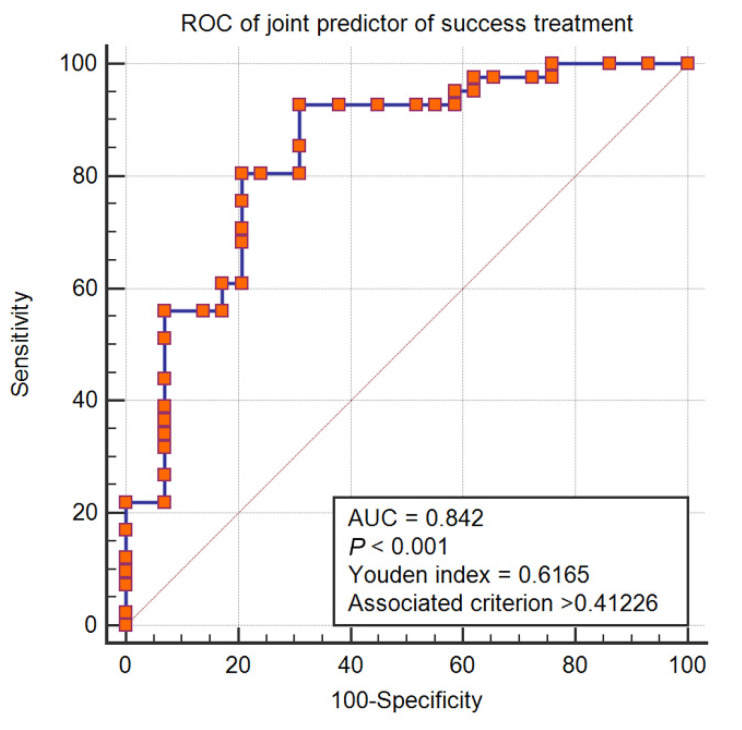
Receiver operating characteristic (ROC) curve of joint predictor of meropenem treatment success.

Based on the aforementioned six confounding factors in adjusted model 2, we drew a receiver operating characteristic curve to investigate the predictive power of the joint predictor (Figure 5) with an area under the curve of 0.842 (95% CI, 0.735–0.918), and a Hosmer–Lemeshow test *p*-value of 0.304. The Youden index was 0.6165, with an associated criterion of >0.4123. The sensitivity and specificity of the ROC were 92.68% and 68.97%, respectively. Furthermore, the final model achieved quite satisfactory performance (AUC = 0.83, recall = 0.97, accuracy = 0.88) in 5-fold cross-validation. All of these results indicate good predictive power.

## 3. Discussion

To the best of our knowledge, this study is the first to show that the meropenem concentration is significantly positively affected by blood urea nitrogen. The multiple linear regression of 210 steady meropenem concentrations we constructed is the first systematic assessment of factors governing the magnitude of steady meropenem concentration in critically ill patients. This approach is more acceptable and readable for clinicians than the classic population pharmacokinetics analysis.

We found that meropenem concentration was significantly affected by renal function indexes. Accordingly, a physiologically based pharmacokinetic study [19] for adult patients with pneumonia also emphasized the ability of renal function indicators to predict meropenem concentration. CG-CLCR was found to be a predictive factor for meropenem concentration in the current study. This observation is similar to the findings of many previous studies [20,21] and conforms to the metabolic characteristics of meropenem. In addition to CL, studies [22,23,24] have shown that serum albumin level also affects the pharmacokinetic parameters of meropenem, in agreement with our results. Although there is no substantial evidence linking blood urea nitrogen and meropenem concentration, we found a significant effect of blood urea nitrogen in our model. Meropenem is a water-soluble antibacterial drug. After intravenous administration, it is mainly distributed in blood and extracellular fluid. The structure of the beta-lactam ring determines its instability. Consistent with most beta-lactams, meropenem is mainly metabolized and excreted by the kidney. The high value of urea nitrogen reflects the impairment of renal function. Therefore, with the weakening of renal function, the concentration of meropenem increased.

Cies J.J. et al. [25] showed that the pharmacokinetic parameters of clearance and the volume of distribution in children were 0.419 L/h/kg and 0.57 L/kg, respectively, while the reported values of elderly people [26] were significantly higher than those of children. Ramon et al. found that age is a potential factor [23] affecting the pharmacokinetics of meropenem. Accordingly, we found that steady meropenem concentration correlated with age. However, the influence of age on drug clearance still needs to be further studied since there is a strong correlation between age and renal function, and it is sometimes hard to identify. In addition to the above factors, studies [13,26,27] showed that body weight also affects the pharmacokinetic parameters of meropenem. However, it was not an independent influencing factor in the present multivariate analysis. This result is consistent with those of previous studies [26] showing no direct influence of age. This is probably because the inter-individual variability of meropenem concentration is largely explained by renal function indexes. The conditions for further reducing the inter-individual variability were more stringent. Moreover, this finding might have been due to relatively centralized patient information.

In a previous study, it was reported that better bactericidal effect can be obtained when 100%T > MIC or even 100%T > 4 MIC is set as the target value in critically ill patients or drug-resistant bacterial infection [28]. However, Zhou Q.T. et al. [22] showed that for elderly patients with lower respiratory tract infections, the %T > MIC of 76% can well predict the clinical success. Additionally, Udy A.A. et al. [29] demonstrated that for patients seriously infected with pathogens with high MIC value, it is even necessary to reach 100% T > 4–5 MIC.

To investigate the target concentration of meropenem to predict its efficacy in patients with pulmonary infections, we assumed that meropenem concentration C_trough_/MIC > 3 was the targeted therapeutic effect 1 and C_trough_/MIC > 4 was the targeted therapeutic effect 2 [30,31,32]. The results of univariate and multivariate logistic regression models demonstrated that C_trough_/MIC > 4 is positively correlated with the presence of the clinical success of meropenem treatment. The result is similar to previous findings [33]. Subjects with C_trough_/MIC > 4 were more likely to have successful meropenem treatment than those with C_trough_/MIC ≤ 4. In contrast, C_trough_/MIC > 3 was not a predictor of meropenem efficacy. Similar study methods have been used to explore its independent predictors of adverse reactions [34]. The result showed that the adverse reactions of meropenem increase when C/MIC > 10 in critically ill patients. However, no adverse events related to meropenem were observed in this prospective study. One reason may be that meropenem had a low incidence of adverse reactions and most of the adverse events can recover spontaneously after drug withdrawal [35,36,37]. The other reason may be that the complex disease characteristics and complicated concomitant drugs among the patients enrolled in the study, which made it more difficult for the doctors to judge the correlation with adverse events. Furthermore, in the Adjust II model, C_trough_/MIC > 4 remained as an independent predictor, indicating that there is an association between C_trough_/MIC > 4 and a favorable outcome and that C_trough_/MIC > 4 is a potential instrument for predicting therapeutic outcomes of meropenem. There are limited clinical PD data, and this study helps to fill this gap, although further studies are necessary to verify the results.

In addition, there are many studies on the effect of infusion duration on the concentration and therapeutic effect of meropenem, but the results are inconsistent at present [38,39,40]. We are also conducting further studies to explore the impact of this factor. In this study, most patients received a dose of 1000 mg q8h (82.81%), so the dose was not taken into account in the final model. Moreover, since only two patients in this study required adjusting the dosing frequency instead of the dose after TDM, the significance of the in-depth analysis of dose effects in this study is limited. Further study can explore dose as an influencing factor in detail. In addition, this study did not conduct in-depth analysis about the drug resistance of meropenem, and our subsequent studies will refer to the latest EUCAT or CLSI M100 implementation standard for antimicrobial sensitivity tests [41,42,43,44] to explore in depth and in detail.

The pathophysiological process of critically ill patients is more complex [45,46], and they exhibit greater inter-individual variability [47]. Blood transfusion, hemodialysis, mechanical ventilation, and hypoproteinemia can affect the absorption, distribution, and metabolism of drugs in the body, thus affecting pharmacodynamics [48]. Therefore, it is necessary to analyze the metabolic process of meropenem in vivo and optimize the dosing regimen in combination with the disease characteristics of patients. As demonstrated, RBC and PCO_2_ were identified as being statistically significant contributors to the likelihood of obtaining a clinical success. It is worth mentioning that, although there was no significant statistical difference in the final model, concomitant drug use was negatively correlated with the presence of clinical success for meropenem treatment. The reason may be that these patients had higher disease severity and worse basic conditions [39]. The antibiotic combination in our study was as high as 73.3%. However, there was no significant statistical difference in the final model of the combined use of other antibiotics on the efficacy. It is necessary to further study the impact of the combined use of antibiotics on the efficacy and safety of treatment, especially whether the combination of antibiotics changes the predicted effect of C/MIC. Some references also suggest that patients with severe infections often had concurrent fungal or virus infections [40,49]. However, analysis is need to further evaluate the basic situation and disease severity of patients with factors in the final model. Moreover, to improve the outcome of meropenem treatment, personalized meropenem dosing needs to be tailored based on TDM results and physiological and biochemical indexes. Our study also helps people who want to recommend TDM to have validated targets [18].

Our study should be continued in the future. In the current study, the number of patients included was limited. MIC values were still not measured at about 30% of the concentration points. This led us not to refine strains for analysis. Moreover, this prospective study only included patients from a single center. Our model will be further validated in a large sample of patients from different regions. It remains to be explored whether the findings are applicable after refining strains.

## 4. Materials and Methods

### 4.1. Patients and Data Collection

The study was performed prospectively in the Second Xiangya Hospital of Central South University. It was approved by the ethics committee of our hospital with the ChiCTR.org Registration number of ChiCTR1900020672. It was conducted from January 2019 to December 2019 and all the participants provided written consent. Inclusion criteria were as follows: We excluded those judged by the clinicians to be unsuitable for the study. Bacterial pneumonia was clinically diagnosed according to the practice guidelines for hospital-associated bacterial pneumonia (HABP)/ventilator-associated bacterial pneumonia (VABP) released by the Infectious Diseases Society of America and the American Thoracic Society [50]. The microbiological methods, biomarkers [51,52], and clinical pulmonary infection scores [53,54] were used. The diagnosis was also based on clinical experience and took the complications of fever, leukocytosis, and an infiltrate on the chest X-ray into consideration. The initial dosage regimen of meropenem was determined according to the prescribed dose. The dose modifications were then adjusted according to clinical response and TDM results by clinicians after ≥48 h of meropenem therapy, during which TDM blood sampling was performed. The dose modifications were made after judging the clinical response and did not affect the judgment of the efficacy outcome. Clinicians either reduced the dose to 500 mg or increased the frequency of administration to achieve the therapeutic effect, based on their treatment experience if necessary. We just recorded the dose adjustment without intervention.

Using a standardized data collection form, we extracted the following information from the electronic medical record information system on the serum sampling day: demographic information, clinical data, laboratory test results, and treatment details. CG-CLCR was calculated using Cockcroft–Gault formula [55,56].

### 4.2. Blood Sampling and Analytical Assays

About 3 blood samples (2–3 mL) on average were collected from each patient. All blood samples were taken after the fifth dose, while trough concentration samples were taken at 30 min before the next dose, and peak concentration blood samples were taken at 2 h after the infusion [20,40,57]. The samples were collected using EDTA-K2 anticoagulant tubes. Blood samples obtained were immediately placed in a refrigerator at 4 °C, transported to the TDM room with an ice pack within 2 hours, and centrifuged for 5 min (3000 rpm). The upper plasma was transferred to the EP tube and stored in −80 °C low-temperature freezer.

We used a fully validated automatic two-dimensional high-performance liquid chromatography technique (Demeter Instrument Co., Ltd., Hunan, China) to measure meropenem concentration. An Aston SNCB (4.6 × 50 mm, 5 µm) column was used for the first-dimensional chromatographic column, and the second was an Aston SBN (4.6 × 200 mm, 5 µm) column. The retention time of meropenem was about 10.46 min, and its specificity was good. The intra-day and inter-day precision were 1.21–2.58% and 0.83–1.80%, respectively. The accuracy of this method was 0.51–1.69%. The extraction recoveries for three concentration levels (high, medium, and low concentrations) were 99.47%, 97.77%, and 97.23%, respectively. This analytical method could be satisfactory for clinical TDM and pharmacokinetic research.

### 4.3. Determinants of Meropenem trough Concentration

To explore the determinants of meropenem trough concentration, we performed univariate analysis, multiple regression analysis, and the diagnosis of the final model. Meropenem trough concentration values were converted to their natural logarithm (ln) before analysis. To perform the model diagnosis, we performed the goodness-of-fit test, the test of linearity, and the evaluation of the residual.

### 4.4. Analysis of Association between Meropenem Concentration and Efficacy

Clinical efficacy assessment of the response to meropenem treatment was based upon clinical response, inflammatory indexes, and chest radiograph. Efficacy of meropenem was classified either as a success or a failure. Persistence or deterioration of clinical response after treatment was considered as clinical failure [34,58], while the remainder were defined as clinical successes. To be more specific, clinical success of treatment was defined as the disappearance of all signs and symptoms related to infection or a marked or moderate reduction in the severity and/or number of signs and symptoms of infection [59].

To investigate the association between meropenem concentration and its efficacy, we performed univariate analysis of three groups of meropenem concentration (trough concentration, 2 h concentration, and peak concentration). Because meropenem is a time-dependent antibiotic and its efficacy is determined by the amount of time the concentration remains above the MIC, we set two targets in combination with MIC. We assumed that meropenem concentration C/MIC > 3 was the targeted therapeutic effect 1 and C/MIC > 4 was the targeted therapeutic effect 2 [60]. T tests, Mann–Whitney U tests, and chi-square tests were used to compare the differences in various indicators between the two efficacy groups. Univariate and multivariate logistic regression analyses were used to ascertain whether target concentration could predict its efficacy.

Respiratory samples for microbiological culture were obtained from endotracheal aspirates (ventilated patients only), bronchoalveolar lavage (BAL) samples, mini-BAL samples, expectorated or induced sputum (nonventilated patients only), or protected brush specimens. The MICs tested by automatic microbial identification and drug sensitivity analysis system using broth microdilution methods directed by the guideline of the Clinical and Laboratory Standards Institute [43].

### 4.5. Statistical Analysis

The continuous variables that conform to normal distribution were expressed as mean ± SD, and those that did not were expressed as median (interquartile range). The normality of quantitative data was tested using the Shapiro–Wilk test. Categorical data were expressed as frequency and rate. A two-sided *p*-value < 0.05 was considered statistically significant. We used Statistical Package for Social Sciences 25.0 (IBM Corp, Armonk, NY, USA), GraphPad Prism 6^®^ software (GraphPad, San Diego CA, USA) and MedCalc version 19 (MedCalc Software Ltd., Ostend, Belgium).

## 5. Conclusions

Blood urea nitrogen, CG-CLCR, and albumin significantly affected meropenem concentration. According to renal function indexes, we can predict possible changes of meropenem concentration and adjust the dosage precisely and individually. C/MIC > 4 is a potential instrument to predict the presence of the successful treatment of meropenem.

## Figures and Tables

**Table 1:** Clinical characteristics of the two treatment groups.

Characteristic	Treatment Success (*n* = 39; 60.9%)	Treatment Failure (*n* = 25; 39.1%)
Male, *n* (%)	30 (76.9%)	17 (68.0%)
Age (years)	64.9 ± 15.2	64.0 (51.0–71.0)
Weight (kg)	61.1 ± 13.0	64.0 (55.5–68.5)
APACHE II score	17.0 (12.0–23.0)	16.0 ± 7.7
Meropenem concentration (*n* = 210, μg/mL)	18.33 (5.92–33.42)	17.47 (7.34–31.99)
C_trough_ (*n* = 101, μg/mL)	8.53 (2.39–22.69)	10.07 (3.53–20.92)
**Concomitant drug use (yes), *n* (%)**		
Antibiotic	27 (69.2%)	21 (84.0%)
Antifungal	16 (41.0%)	13 (52.0%)
Antiviral drug	12 (30.8%)	14 (56.0%)
**Physiological and biochemical indexes**		
PO_2_ (mmHg)	67.2 (57.1–83.8)	67.7 (58.3–85.9)
PCO_2_ (mmHg)	35.0 (28.9–53.4)	38.0 (34.5–50.0)
[Na^+^] (mmol/L)	135 ± 6.7	135 (131–141)
[Cl^−^] (mmol/L)	106 ± 8.3	107 (101–110)
Hemoglobin (g/L)	105 ± 26	99 ± 25
Red blood cells (10^12^/L)	3.5 ± 0.9	3.3 ± 0.9
Platelets (10^9^/L)	166 (130–333)	175 ± 123
Alanine transaminase (U/L)	32.5 (20.3–53.2)	35.1 (11.9–57.8)
Aspartate aminotransferase (U/L)	40.0 (26.7–65.8)	50.5 (33.5–91.9)
Albumin (g/L)	27.1 ± 5.0	26.9 ± 3.6
Total bile acid (μmol/L)	5.2 (3.5–12.9)	5.4 (2.2–10.1)
Blood urea nitrogen (mmol/L)	7.81 (5.19–15.96)	9.46 (5.96–17.13)
Creatinine (μmol/L)	66.7 (54.4–154.4)	80.8 (52.7–164.7)
Uric acid (μmol/L)	175.8 (112.6–345.7)	188.6 (146.6–346.9)
CG-CLCR (mL/min)	76.9 (40.2–100.9)	74.2 (40.3–100.2)
**Inflammatory indicators**		
Procalcitonin (μg/L)	0.69 (0.14–4.08)	0.32 (0.15–3.13)
C-reactive protein (mg/L)	124.0 (93.0–214.0)	124.0 (30.5–245.0)
Erythrocyte sedimentation rate (mm/h)	74 ± 40	70 ± 40
Temperature (°C)	38.0 ± 1.0	38.2 ± 0.8
**C/MIC > 4 (yes), *n* (%, *n* = 139)**	55 (68.8%, 55/80)	23 (39.0%, 23/59)
**C_trough_/MIC > 4 (yes), *n* (%, *n* = 70)**	20 (48.8%, 20/41)	6 (20.7%, 6/29)
**MIC (yes), *n* (%, *n* = 70)**		
≤1	14 (34.1%, 14/41)	1 (3.4%, 1/29)
2	12 (29.3%, 12/41)	4 (13.8%, 4/29)
≥8	15 (36.6%, 15/41)	24 (82.8%, 24/29)

The normality of quantitative data was analyzed by Shapiro–Wilk normal test; the non-normal distribution data were presented by median (IQR), while the normal distribution was presented by mean ± SD; and C, MIC, C/MIC, and C_trough_/MIC were displayed by meropenem concentration points. The number of overall concentrations, C/MIC, trough concentrations, and C_trough_/MIC are 210, 139, 101, 70, respectively. APACHE, Acute Physiology and Chronic Health Evaluation; CG-CLCR, endogenous creatinine clearance rate; IQR, interquartile range; SD, standard deviation; [Cl^−^], serum chloride concentration; [Na^+^], serum sodium concentration.

**Table 2:** Correlation analysis of ln (C_trough_).

Variable	Coefficient Index	*p*-Value
Gender	−0.192	0.055
Age	0.388 **	<0.001
Weight	−0.147	0.143
APACHE II score	0.086	0.391
dose	−0.289 **	0.003
infusion duration	0.207 *	0.038
**Concomitant drug use (yes), *n* (%)**		
Antibiotic	0.034	0.735
Antifungal	0.424 **	<0.001
Antiviral drug	0.110	0.275
**Physiological and biochemical indexes**		
PO_2_	0.070	0.490
PCO_2_	−0.140	0.164
[Na^+^]	0.402 **	<0.001
[Cl^−^]	0.093	0.353
Hemoglobin	−0.429 **	<0.001
Red blood cells	−0.416 **	<0.001
Platelets	−0.040	0.694
Alanine transaminase	−0.110	0.274
Aspartate aminotransferase	0.126	0.211
Albumin	0.237 *	0.017
Total bile acid	−0.010	0.918
Blood urea nitrogen	0.606 **	<0.001
Creatinine	0.548 **	<0.001
Uric acid	0.560 **	<0.001
CG-CLCR	−0.694 **	<0.001
**Inflammatory indicators**		
Procalcitonin	0.332 **	0.001
C-reactive protein	0.020	0.841
Erythrocyte sedimentation rate	0.206 *	0.038
Temperature	0.037	0.713

* The variables are significant at the level of 0.05 (double tail); ** the distinction was statistically significant at the level of 0.01 (double tail).

**Table 3:** Multiple linear regression analysis of meropenem concentration determinants.

Variable	Coefficient	Standardized Coefficient	T	*p*-Value	VIF
Blood urea nitrogen	0.051	0.390	4.820	<0.001	1.396
CG−CLCR	−0.009	−0.386	−4.801	<0.001	1.382
Albumin	0.071	0.232	3.243	0.002	1.088
Infusion duration	0.495	0.195	2.839	0.006	1.008
Constant value	−0.914		−1.154	0.251	
F		29.360			
*p*		<0.001			
R^2^		0.531			

Dependent variable: ln (C_trough_).

## Data Availability

I declare that my research data is available. I will share my research data with other researchers if they need it. Specifically, the data will include (but are not limited to): raw data, processed data, software, and algorithms. If additional files are required, they will also be shared on request. Meanwhile, the data will become available from the date it is published. Researchers can email me if they are interested in the study and need the research data for analysis.
